# Health Personality, Consumer Health Activation, and Loneliness

**DOI:** 10.1093/geroni/igaa057.1247

**Published:** 2020-12-16

**Authors:** Nicholas Cone, Peter Martin

**Affiliations:** Iowa State University, Ames, Iowa, United States

## Abstract

The purpose of this study was to identify relationships between health personality traits, consumer health activation (CHAI) and loneliness. Data for these analyses were collected by a large provider of Medicare Supplemental Health Insurance. The study consisted of 3,907 participants, 65 years and older. Participants were surveyed on health personality (e.g., Health Neuroticism, Health Extraversion, Health Openness, Health Agreeableness, and Health Conscientiousness), Consumer Health Activation, and Loneliness. Structural equation modeling and mediation were conducted through Mplus. The hypothesized model fit without direct paths from health personality to loneliness was not optimal. Adding direct paths from health neuroticism, health openness, and health agreeableness to loneliness resulted in an excellent fit, □2 (5) = 0.86, RMSEA = 0.00, CFI = 1.00. Health neuroticism and health openness were negatively related to health activation, which suggests respondents were less likely to be active about their health. Alternatively, health agreeableness and health conscientiousness were positively related to health activation, indicating more health activation. Mediation was tested for pathways from health personality dispositions to loneliness through health activation. The results suggest individuals higher in health neuroticism or health openness were less activated, which in turn indicated higher loneliness. Moreover, those higher in health agreeableness or health conscientiousness were more activated and indicated less loneliness. This study provides an understanding about loneliness through health personality and health activation. Future research should explore interventions for older adults with specific health personalities, or health activation to reduce loneliness levels.

